# Characteristics, availability and uses of vital registration and other mortality data sources in post-democracy South Africa

**DOI:** 10.3402/gha.v5i0.19263

**Published:** 2012-12-27

**Authors:** Jané Joubert, Chalapati Rao, Debbie Bradshaw, Rob E. Dorrington, Theo Vos, Alan D. Lopez

**Affiliations:** 1 School of Population Health, University of Queensland, Herston, QLD, Australia; 2 Burden of Disease Research Unit, South African Medical Research Council, Tygerberg, South Africa; 3 Centre for Actuarial Research, University of Cape Town, Cape Town, South Africa

**Keywords:** mortality data sources, South Africa, civil registration, census, surveys, surveillance, data availability

## Abstract

The value of good-quality mortality data for public health is widely acknowledged. While effective civil registration systems remains the ‘gold standard’ source for continuous mortality measurement, less than 25% of deaths are registered in most African countries. Alternative data collection systems can provide mortality data to complement those from civil registration, given an understanding of data source characteristics and data quality. We aim to document mortality data sources in post-democracy South Africa; to report on availability, limitations, strengths, and possible complementary uses of the data; and to make recommendations for improved data for mortality measurement. Civil registration and alternative mortality data collection systems, data availability, and complementary uses were assessed by reviewing blank questionnaires, death notification forms, death data capture sheets, and patient cards; legislation; electronic data archives and databases; and related information in scientific journals, research reports, statistical releases, government reports and books. Recent transformation has enhanced civil registration and official mortality data availability. Additionally, a range of mortality data items are available in three population censuses, three demographic surveillance systems, and a number of national surveys, mortality audits, and disease notification programmes. Child and adult mortality items were found in all national data sources, and maternal mortality items in most. Detailed cause-of-death data are available from civil registration and demographic surveillance. In a continent often reported as lacking the basic data to infer levels, patterns and trends of mortality, there is evidence of substantial improvement in South Africa in the availability of data for mortality assessment. Mortality data sources are many and varied, providing opportunity for comparing results and improved public health planning. However, more can and must be done to improve mortality measurement by improving data quality, triangulating data, and expanding analytic capacity. Cause data, in particular, must be improved.

The importance of mortality data for public health and development is widely acknowledged and publicly recognised since the 1600s in Graunt's Bills of Mortality (1). Globally, the importance of this continues to be reflected in a number of initiatives, such as the inclusion of mortality as a component of the Human Development Index (HDI) (2) and the expression of Millennium Development Goals (MDGs) 4, 5, and 6 in terms of mortality (3, 4). Although global, these indicators and targets firstly need to be measured at national and sub-national levels. However, the required data for their measurement are often not readily available, particularly in developing countries and the world's poorest populations. In 2007, the *Lancet* “Who Counts?” series confirmed that few countries in greatest need of vital-event and cause-of-death data have the capacity to obtain these (5).

Civil registration systems, population censuses, and surveys are highlighted as principal sources for measuring mortality (6–8). Recent reports also include sample vital registration and demographic surveillance systems (9). Civil registration with high coverage and accurate medical certification and coding of the cause of death remains the ‘gold standard’ source of continuous mortality data. However, only approximately 30% of the world's population live in areas with >90% completeness of death registration (10). In most African countries, for example, less than one-quarter of deaths are registered (11). Only 2% of the countries in Africa and South-East Asia have complete death registration data and half of the countries in these regions record no cause-of-death data (5). Other challenges include incomplete birth registration, misreported age at death, delays of death records under medico-legal investigation, and delays in releasing the data. For cause-of-death data, limitations include omission of the cause on the death certificate, inclusion of a cause, but not certified by a physician, inclusion of a physician-certified cause, but recorded as ‘ill-defined’ or ‘undetermined’, or a physician-certified cause, but inadvertently misdiagnosed or advertently euphemised or misclassified.

In such settings, mortality researchers rely on available alternative data collection systems such as censuses, surveys, and sample and small-area systems (9). Censuses provide essential data on population denominators and the opportunity to collect national mortality data through direct recall of household events in defined time periods, or indirect measures by collecting information on parental, spousal, sibling or child survival. Accurate information can provide estimates of childhood and adult mortality by population sub-groups and small areas. Potential weaknesses include: recall bias, event omission due to population undercount, household collapse, or mother's death, question limitations due to the length of the questionnaire, inaccurate age reporting, and the relative infrequency of enumerations at 5- or 10-year intervals. Household surveys have played a significant role over the past four decades in assessing childhood mortality through birth histories in areas with inadequate civil registration (12). Such surveys can also inform adult mortality assessment through questions on parental, sibling and spousal survival, and questions about household deaths in a specified period. Household surveys have the advantage that they can be carefully monitored for data quality, but can also be compromised by insufficient fieldwork training and supervision. Further limitations include the inability to make estimates for areas below provincial/state level, recall bias, and missing births and deaths due to discord- or death-related household disintegration or zero-survivor families (11).

Health and demographic surveillance sites (HDSSs) in defined geographic area, such as those co-ordinated by the International Network for the Demographic Evaluation of Populations and their Health in Developing Countries (INDEPTH), constitute another approach to collecting mortality data and fills an important gap in mortality assessment in many low- and middle-income countries (13). Births and deaths information are critical in each HDSS, actively identified through regular visits by trained fieldwork staff to all households in the HDSS, recording events in the period since the previous visit. With most deaths in these sites not occurring in health facilities, the cause of death is typically assessed via a verbal autopsy instrument and interviews with close kin or carers of the deceased. This is commonly followed by physician assessment and consensus acknowledgement of the probable cause of death. As a promising alternative, computerised procedures have been developed with Bayesian probabilistic modelling over the past decade, leading to the InterVA suite of models and culminating with the recent, freely available InterVA-4 model (14). Though typically intensive and thorough in pursuing vital data, and usually achieving complete, or close to complete, death recording, HDSSs are generally restricted to small geographic areas and their populations are not necessarily representative of the national population. The lay report of the circumstances leading to death, often reported a few months after the event, can be a further limitation.

While civil registration systems remain under-developed in most African countries, recent political and public services transformation in South Africa, along with determined efforts by researchers, have focussed on enhancing the civil registration system and advancing mortality data availability from vital statistics compilation (5, 15). Additionally, mortality data items have been included in local censuses, national surveys, HDSS data collections, and condition-specific registry, disease notification and mortality audit programmes. Each of these sources has relative strengths and limitations, and an understanding of the characteristics of these sources and the quality of the data produced by them is important to guide the utilisation of their data and exploit potential complementary properties. As South African cause-specific mortality patterns have been used to model mortality in many sub-Saharan Africa countries (16, 17), a closer look at the availability and quality of such data may be useful beyond its local value.

This article aims to review civil registration and alternative data collection systems for mortality estimation in post-democracy South Africa. Our objectives are to document these data sources, report on public availability and possible complementary uses of data from these sources, discuss data limitations and strengths in the context of their use for particular mortality indicators and analyses, and make recommendations for improving data quality and the reliability of mortality estimation.

## Study design

Background information of mortality data sources, data availability, and possible complementary uses of mortality data were assessed by reviewing blank survey and census questionnaires, death notification forms, death data capture sheets, and patient treatment and clinic/hospital cards; legislation; electronic data archives and databases and web-based data repositories; and related information in scientific journals, research reports, statistical releases, government reports, working papers, and books.

In the context of the Africa Programme on Accelerated Improvement of Civil Registration and Vital Statistics (APAI-CRVS) (18), and the global importance attached to civil registration systems for mortality assessment (19), the review includes a particular focus on the development of the country's civil registration and vital statistics system over the past century. Additionally, summary background information was extracted and data availability assessed for three post-democracy population censuses, a number of national household surveys, one rapid mortality surveillance system, and three HDSSs. Tuberculosis, cancer, and injuries are major contributors to the country's mortality burden, and key information was obtained of three facility-based surveillance systems related to these causes. Additionally, given the international pressure to provide reliable information about child and maternal health to inform MDGs 4 and 5, and the potential to complement vital registration data, summary information was extracted about three facility-based mortality audit programmes for peri-natal, child and maternal deaths.

## Mortality data sources in South Africa

### Civil registration and vital statistics systems in South Africa: a century of challenges

Starting in 1867, a series of laws on birth and death registration were enacted at sub-national level. Half a century later, the Births, Deaths and Marriages Registration Act of 1923 promised the possibility of uniform registration practice across the country (20–23). The act remained in place until replaced by the Births and Deaths Registration Act of 1992 that, unlike the 1923 act, required complete coverage of all people and all geographic areas (24). Between 1923 and 1992, however, the civil registration and vital statistics systems faced numerous challenges. Neither the single, national act of 1923, nor the establishment of the national statistical office in 1914 brought about an inclusive, comprehensive civil registration system. Instead, it was a partial registration system, covering selected segments of an ideologically contrived population, based on ‘homeland’/common land, rural/urban, and population-group differentiation.

Over time, geographic and population fragmentation became further entrenched, formalised by legislation, significantly stunting the civil registration system through the under-registration of deaths in large parts of particularly the majority, Black African,^1^ population group. Such legislation included the restriction of land leasing and ownership among Black Africans to ‘homelands’ or designated reserves, mainly in under-developed rural areas with limited economic possibilities (25), and dividing the non-‘homeland’ or common area into freely accessible (rural) and restricted (urban) areas. This residence and movement control confined the majority of Black Africans to rural residence. With the Bantu Homelands Citizenship Act of 1979, Black Africans were required to become a citizen of one of 10 ‘homelands’ (22, 23, 26). After granting independence to four ‘homelands’, they became excluded from the South African statistical system and responsible for generating their own vital registration information (21). However, these states were largely incapable of doing this (27) and, in the remaining six ‘homelands’, several laws and circumstances constrained civil registration (22).

The Population Registration Act of 1950 made provision for the compilation of a manual population register that, counter-intuitively, played a minor role in producing vital statistics (22, 23). In 1972, a computerised population register was initiated, but did not capture the civil details of Black Africans until 1986 (21, 22). The civil registration was also affected by the 1923 act under which death registration for rural-living Black Africans was voluntary (20, 21), implying that during the 1920s approximately 86% of Black Africans (28) were under no obligation to register any death. Medical certificates were required for urban but not rural deaths (20), further inhibiting vital statistics in South Africa.

In addition to these inhibiting effects and the lack of registration infrastructure and resources in most of the ‘homeland’ areas, reporting of vital events was also probably restrained by the many disruptive effects of forced removal and resettlement (25, 29–32) on people's motivation and means to do so (25, 31, 33). Moreover, legislation, including civil registration legislation, itself impeded vital registration for decades as one act ensured that, for the majority population, rural living was compulsory while, at the same time, another ensured that rural death registration was not (21). With completeness of Black African death registration estimated to have ranged from about a quarter of all deaths during the late 1960s to about half in the mid-1980s (34), the vital-event details of this group in particular became severely under-represented in vital statistics, limiting the use and generalisability of civil registration data considerably. (21, 22), Thus, although official mortality statistics have been collated and published since 1910 (20), the above events led to large biases in vital statistics data, and numerous barriers to producing reliable, representative and timely mortality statistics.

#### Transformation during the 1990s affecting civil registration

During the 1990s, however, under a new, democratic dispensation, major and rapid political and social transformation ensued in all spheres of governance, including a commitment to transforming the civil registration and vital statistics systems into an information system that effectively serve civil record-keeping and public health planning. Bah (23) identified three key events during the 1990s which held new promise for vital registration coverage and content: 1) the passing of the Births and Deaths Registration Act of 1992 (24), leaving no scope for optional or differential registration; 2) the adoption of the interim Constitution of South Africa in 1993 (35), ensuring the consolidation of all geographic segments into one geo-political unit and, therewith, the centralisation of the civil registration system under one entity; and 3) the agreement among three key role players – the Department of Health, Department of Home Affairs (DHA), and the official national statistical agency, Statistics South Africa (Stats SA) – to establish, in collaboration with health researchers, a joint technical committee tasked to enhance civil registration and improve the vital statistics system (23, 36). These events spawned a range of initiatives to increase the registration of deaths and improve the quality of vital-event data (23, 37, 38), including the introduction of a new death notification form to bring local data in line with international standards and to achieve compliance with WHO standards for the certification of causes of death (36, 39, 40).

Institutional capacity was strengthened through study tours and visits of key government officials to civil registration/vital statistics offices in Australia, Sweden, and the United States (23). More capacity and initiatives have been developed to enhance coverage of death registration, including the establishment of provincial task teams who developed a strategy to enhance registration, and facilitated the introduction of the new death notification form to ensure its implementation; distribution of certification and ICD code manuals to hospitals and health professional and academic organisations for staff training; letters to all registered doctors, informing them of the new procedures and relevant guidelines; the development of guidelines by the Department of Health on birth and death registration; training of health workers in all provinces about the importance and process of vital registration; making the necessary forms for birth registration available to mothers at the time of delivery; and assisting mothers to complete and submit the forms to the DHA (21, 36, 41).

#### Capturing the effects of transformation in completeness estimation

These specific efforts, along with the political and social transformations mentioned previously, are likely to have contributed to the increasing levels of completeness of registration for both adult and child deaths over the past two decades. Estimation of completeness of registration during the 1980–90s was a very complicated task. However, meticulous, comprehensive research since the late 1980s has carefully sought to understand the merit of different local mortality data sources and the applicability of different indirect methods in the South African mortality data context over time (27, 42–44). Application of the Bennett and Horiuchi's Synthetic Extinct Generations method (45), for example, to deaths from Stats SA's vital registration database and the Population Register, relative to population estimates from the ASSA600 AIDS and Demographic Model of the Actuarial Society of South Africa, estimated that the level of adult death registration improved from 85% to about 90% for the period 1996–2000 (43).

For the period 1996–2006, the estimated level of death registration improved from 43 to 89% for infants; from 44 to 78% for children under 5 years; and from 43 to 57% for children aged 1–4 years (46). These childhood estimates were derived using a multi-stage method described in Darikwa and Dorrington (46), using registered death data from civil registration; data from the 2007 Community Survey (child deaths over the past 12 months as reported by households, children ever born/children surviving data, and data on the survival of the last child born to women aged 12–49 years); 2001 Census (reported household deaths); and data from previous research (47–50) based on the 1998 South Africa Demographic and Health Survey (SADHS) and 1996 Census. Completeness levels between 1996 and 2006 were determined by assuming that the completeness of death registration follows a logistic trend over time; that completeness in any age group did not decrease over the period; and that the trend in completeness is smooth (i.e. fluctuations in the data are not due to fluctuations in completeness). A logistic curve was then fitted (46, 51).

The completeness in infant death registration has increased particularly rapidly since 2001, most probably resulting from the government's intensified efforts to register births and deaths, particularly in facilities, along with many infant deaths occurring before leaving these facilities. This bodes well for more accurate estimates of this important indicator, in particular the prospect of deriving infant mortality directly from vital registration data with minimal adjustment (46).

#### Mortality data and data availability from civil registration

Death notification forms are administrated by DHA. These forms are then processed by Stats SA to capture the mortality and selected socio-demographic and health data (52). From this, cause-specific mortality statistics are produced by Stats SA, coded to three-digit codes according to the tenth revision of the International Statistical Classification of Diseases and Related Health Problems (ICD-10) (53). Anonymous unit record cause data for 1997–2009 are available electronically upon request, subject to a data user's agreement. To protect the identity of the deceased, certain fields such as the date of death and place of death or residence, are not publicly available. Mortality and cause-of-death data for 2006–2008 are additionally freely available in web-based data repositories, and users can analyse and tabulate a number of variables with socio-demographic (e.g. age and sex), health status (e.g. smoking and pregnancy status), and cause data (e.g. immediate and underlying cause) with online and available software Nesstar and SuperWEB (54). Reports (online or hard copy) from 1997 to 2009 offer a variety of tabulations for all-cause and cause-specific mortality, both nationally and provincially (55). The latest reports additionally offer tabulated numbers of death and selected cause patterns by district municipality (55). Over the past decade, the production of official mortality statistics has improved considerably, and mortality data are available annually, reporting on deaths that occurred in the calendar year 2 years prior to publication.

### National surveillance, censuses and surveys

#### Rapid Mortality Surveillance System using national data from the Population Register

The 1990s, still suffering the effects of the pre-democratic civil registration and vital statistics system, presented with a substantial time lag in the production and release of mortality data. These delays were particularly problematic amidst the rapid unfolding of an enormous HIV/AIDS epidemic and the perceived significant mortality changes in the population. An urgent need thus arose for more up-to-date mortality statistics and the continuous monitoring of more recent mortality trends. In response, the South African Medical Research Council (MRC) in collaboration with the University of Cape Town, negotiated access to death data by age and sex as recorded on the National Population Register maintained by the DHA. A project was set up at the MRC in 1999 to capture and monitor trends in these data as rapidly as possible. The MRC database is updated monthly with death data provided electronically by the DHA, allowing mortality by age and sex to be monitored within a few months after the date of death (56, 57).

As for the vital statistics system of Stats SA, the source of the Rapid Mortality Surveillance System (RMS) is death notifications submitted to the DHA. However, there is a difference between the numbers of deaths captured by these two systems. Stats SA captures all deaths notified to the DHA, while the RMS only captures those deaths notified to the DHA which have been recorded onto the National Population Register, i.e. only the deaths of individuals with a South African birth or identity certificate (as only people with these certificates are on the Population Register). The RMS, therefore, captures fewer deaths compared to Stats SA's vital statistics system, on average about 12% less for the years 2002–2009, but more than sufficient numbers to serve the purpose for which it was developed (on average 493,000 deaths for 2000–2011) (57).

While mortality reports currently are being published with a time lag of approximately two years, the RMS remains useful for providing information about deaths within months after occurrence. Additionally, the RMS is useful for tracking changes in mortality due to the roll out of interventions such as programmes to prevent mother-to-child transmission of HIV, and provision of antiretroviral therapy.

The RMS data are received and stored by the SA MRC for continuous rapid assessment of changing trends in the deaths by age and sex. The availability of the data has been negotiated with the purpose of rapidly assessing and informing about changes as assessed by experienced mortality researchers. To inform research and policy action adequately, the data needs to be interpreted taking into account the prevailing levels of completeness of death registration, the extent of birth and death registration into the population register, and levels of population growth. Findings from the rapid mortality surveillance system are regularly reported in publicly available papers, reports, and conference presentations (43, 56–59).

#### Post-democracy population censuses: 1996, 2001 and 2011

Post-unification (1910) and pre-democracy (1994), 14 population censuses with variable coverage have been conducted in South Africa, the first in 1911. Since democracy, three censuses that covered the total South African population have been conducted, respectively, in 1996, 2001, and 2011. For all three censuses, a post-enumeration survey was undertaken to determine the degree of under- or over-count in the population. For the 1996 and 2001 censuses, the population was undercounted by an estimated 10.7% (60) and 17.6% (61), respectively. Results from the 2011 census have not been published yet. More information is available at the Population Statistics Section of the Stats SA website (62).

#### Mortality data from censuses and data availability

Information about the types of census data collected to measure mortality in different interest groups, and the years for which such data were collected, are shown in Table 1. Census data tabulations at national and sub-national level are available on request from Stats SA. Children ever born/children surviving (CEB/CS) and parental survival data from a 10% sample of the household unit record data for the 1996 and 2001 censuses are available together with selected socio-demographic variables in web-based data repositories via Nesstar and SuperWEB (54). For 2001, deaths reported by households are also available in the 10% sample. Metadata for the census variables are available on Stats SA's 1996 and 2001 census web pages (62). Additional data input is required to calculate adult and child mortality rates from these variables. Stats SA commissioned the Centre for Actuarial Research to analyse and evaluate the mortality data collected in the 2001 census. The resultant detailed report (44) contains essential information for users on the quality of child and adult mortality data collected.

**Table 1:** Post-democracy data sources for mortality analysis in South Africa by enumeration years

	Enumeration year(s)			
	
Data sources	Child mortality	Adult mortality	Maternal mortality	Causes of death
Vital Registration (VR)	1997–current*	1997–current*	1997–current*	1997–current*,††
Rapid Mortality Surveillance System (RMS)	1998–current*	1998–current*	–	1998–current ‡‡
Population census	1996,† 2001,† 2011†	1996,∣∣ 2001,∣∣,¶ 2011∣∣,¶	2001 & 2011**	2001 & 2011‡‡
Demographic Surveillance Sites (DSS): >Agincourt	1992–current*	1992–current*	1992–current*	1991–current§§
>ACDIS	2000–current*	2000–current*	2000–current*	2000–current§§
>Dikgale	1996–current*	1996–current*	1996–current*	2011–current§§
Community Survey (CS)	2007‡	2007‡	2007‡	2007‡, ‡‡
October Household Survey (OHS)	1993–1999‡	1993–1999‡	–	1993–1998‡, ‡‡
General Household Survey (GHS)	2002‡	2002–2011‡	–	–
Demographic & Health Survey (DHS)	1998;‡ 2003‡	1998 & 2003‡	1998 & 2003‡	1998 & 2003‡, ‡‡
National Income Dynamics Study (NIDS)	2008‡	2008‡	–	2008‡, ‡‡

Source: Table created by authors from vital registration and survey information as follows: VR: Stats SA, 2012 (54), Stats SA, various years (55); Census 1996: Stats SA, 2012 (88); Census 2001: Stats SA, 2012 (54); OHS: National Research Foundation (63); GHS and CS: Stats SA web-based Nesstar information and Stats SA electronic reports (54, 69, 89); DHS: Department of Health *et al*., 2002 (47), Department of Health *et al*., 2007 (68); NIDS: Moultrie & Dorrington, 2009 (90).

* Notes: Direct estimation from routine surveillance

† children ever born/children surviving (CEB/CS)

‡ See Table 2

∣∣ parental survival

¶ deaths in the household

** deaths in the household plus pregnancy/delivery-related question

†† cause obtained from medical certificate of cause of death on death notification form (BI-1663), or headman reporting on death report (BI-1680)

‡‡ censuses and surveys are not traditional ways to collect cause-of-death data. For the censuses, surveys and RMS, causes were broadly indicated as natural/unnatural, pregnancy/delivery-related, or accident/violence-related causes

§§ cause ascertained via information from a verbal autopsy instrument.

#### Post-democracy national surveys

Brief background information of five national surveys is individually given below after which mortality data from these surveys and its availability for public use are discussed collectively. Details about the enumerated number of households and participants, and the types of mortality data collected, are in Tables 1 and 2.

**Table 2:** National surveys measuring mortality, by year of survey, number of households and persons enumerated, and different methods of mortality measurement

Year	Number of households	Number of persons	Deaths in the household	Parental survival	Sibling survival	Spousal survival	Full birth histories	Summary data on births, deaths of previous births, and surviving children
October Household Survey (OHS)								
1993	30,233	136,468	✓ 12 months					✓W egb15–49†
1994	30,279	132,469	✓ 12 months					✓ W egb <55†
1995	29,700	130,787	✓ 22 months	✓				✓ W egb <55†
1996	15,917	72,889	✓ 22 months	✓				✓ W egb <55†
1997	29,810	140,015	✓ 22 months	✓	✓ (Sisterhd)*	✓		✓ W egb†
1998	18,981	82,262	✓ 22 months	✓	✓ (Sisterhd)*	✓		✓ W egb†
1999	26,164	106,650	✓ 12 months					✓ (W gb12mo)‡
								
General Household Survey (GHS)								
2002	26,243	102,461		✓				✓ (W 12–49)
2003 to 2011	Varied: 24,333 to 29,236	Varied: 94,263 to 109,824		✓				
								
Community Survey (CS)								
2007	246,618	949,105	✓ 12 months	✓				✓ (W 12–49)
								
Demographic and Health Survey (DHS)								
1998	12,540	17,500	✓ 12 months	✓	✓		✓	✓ (W 15–49)
2003	7,756	18,274		✓	✓		✓	✓ (W 15–49)
								
National Income Dynamics Study (NIDS)												
2008	7,305	28,255	✓24 months	✓			✓	✓ (W 15–49)

Source: Table created by authors from the following information on surveys: OHS: National Research Foundation (63); GHS and CS: Stats SA web-based Nesstar information and Stats SA electronic reports (54, 69, 89); DHS: Department of Health *et al*., 2002 (47), Department of Health *et al*., 2007 (68); NIDS: Moultrie & Dorrington, 2009 (90), Leibbrant *et al*., 2009 (71).

* Notes: sisterhood method;

† W egb: women who have ever given birth;

‡ W gb12mo: all women who have given birth in the last 12 months.

#### October Household Surveys: 1993–1999

The establishment of the October Household Surveys (OHS) programme in 1993 marked the beginning of the national collection of demographic information on an annual basis. The OHS was a cross-sectional sample survey undertaken by Stats SA from 1993 to 1999, aiming to collect individual and household information that covered a range of development and poverty indicators. The OHS was replaced by the General Household Survey. The surveys were based on a probability sample of a large number of households, targeting residents in private households and workers–hostels countrywide. Fieldworkers visited sampled households and filled the survey questionnaire during face-to-face interviews (63). See Tables 1 and 2 and the Stats SA (64) and University of Cape Town's DataFirst (65) websites for more information.

#### General Household Survey: 2002–2012

The General Household Survey (GHS) has been conducted annually by Stats SA from 2002 to 2011 and was in the field until September 2012 for the next round. The GHS was instituted to monitor development indicators and development programmes on a regular basis. The survey aims to measure multiple facets of the living conditions of the country's households, and the quality of service delivery in selected service sectors. The GHS is a cross-sectional survey, based on a representative sample drawn from the total population. The target population is private households and residents in workers–hostels. Using probability-proportional-to-size principles, a multi-stage, stratified random sample is drawn. Households are visited by fieldwork teams and an extensive questionnaire is filled by enumerators during face-to-face interviews (66, 67). Further information is available in Tables 1 and 2, and at the Stats SA (64) and DataFirst websites (65).

#### South Africa Demographic and Health Survey: 1998 and 2003

Post-democracy, two national Demographic and Health Surveys (DHSs) were conducted collaboratively by the Department of Health, SA MRC, and OrcMacro. The 1998 SADHS employed a two-stage sample based on 1996 census demarcations and stratified according to the nine provinces, each subsequently stratified by urban/non-urban residence (47). The 2003 survey sample, based on the enumeration areas created during the 2001 census, was designed as a nationally representative sample of households. Stratification took place according to the provinces and subsequently by urban/non-urban residence (68). Eligible women were prompted for full birth histories in both surveys. Tables 1 and 2 highlight more information about the types of mortality data collected. More information about the 1998 and 2003 surveys is available in the final full reports (47, 68).

#### Community Survey: 2007

The Community Survey (CS), conducted by Stats SA, was a large-scale nationally representative inter-census household survey conducted in 2007, designed to provide information on the trends of selected demographic, social, and socio-economic profiles of the population. The sampling procedure included a two-stage stratified random sampling process, the first involving the selection of enumerator areas within each municipality, and the second the selection of dwelling units within enumerator areas. Enumerators visited the selected sampled dwelling units and completed questionnaires during face-to-face interviews with study participants (69). The realised sample was adjusted to replicate the national population in a way that the data are consistent internally and with other censuses and surveys (70). Tables 1 and 2 and the Stats SA (64) and DataFirst (65) websites hold more information.

#### National Income Dynamics Study, Wave 1: 2008

The National Income Dynamics Study (NIDS) was South Africa's first national panel study to document the dynamic structure of households and changes in the incomes, expenditures, assets, access to services, education, health, and well-being of household members. The target population was private households in all provinces and residents in workers–hostels, convents, and monasteries. Households were sampled with a stratified, two-stage cluster sample design, randomly selecting 400 primary sampling units in the first stage from Stats SA's 2003 master sample. In each primary sampling unit, two dwelling-unit clusters were selected. The first fieldwork wave commenced in February 2008. Information was collected on all household members, both resident and non-resident. A household questionnaire and an individual questionnaire for each adult and child in the household were administered via face-to-face interviews in each household (71). See Tables 1 and 2 and the NIDS methodology report (71) for more information.

#### Mortality data collected in national surveys and data availability

Mortality was assessed for different age or interest groups across the surveys, and mortality items sometimes varied within surveys across time (Tables 1–2). Items to assess mortality via both direct and indirect measures were included in these surveys. Assessments of deaths in the household, parental survival, and summary birth histories were included most frequently across time and surveys. When a death was reported, selected further information about the death and deceased were collected. The years of enumeration, interest group, and mortality data items used are shown in Tables 1 and 2.

Unit record data from the OHS can be requested for use in the DataFirst Research Data Centre at the University of Cape Town. The Research Data Centre makes data, statistical analysis software, and trained staff available, free of charge, for this purpose (65). OHS data and metadata are available on compact disc for a fee from Stats SA (72). Unit record data from the GHS and CS are available on disc from Stats SA via request. Stats SA's web-based data repositories (54) contain information about mortality as collected in the GHS and CS. From the GHS, parental survival data for 2002–2010 can be found via SuperWEB, and for 2002–2008 via Nesstar (54). From the CS, parental survival data are available via Nesstar and SuperWEB, and summary birth histories and death-in-the-household data via Nesstar (54). Users can tabulate CS death-in-the-household data by selected demographic variables (e.g. age and sex) and sub-national entities (e.g. province and district council). Stats SA also responds to special requests for tabulations from the Stats SA surveys without requiring a data user's agreement. Due to the poor quality of the data, the 2003 DHS failed to provide reliable estimates of adult and maternal mortality from the parental and sibling survival questions (68). The birth history data, however, were used to estimate levels and trends in infant and child mortality, and apart from a chapter presenting these results (68), unit record data from the 1998 and 2003 DHS surveys are available per request and by signing a data user's agreement, from the Department of Health and SA MRC. For NIDS, data and supportive documentation for Wave 1 are available via DataFirst servers upon completion of an online form and agreement to the terms of data use (71). Publicly available datasets from any of these surveys contain only non-confidential data.

Extensive resources and information related to these surveys, including questionnaires, reports, metadata, code lists, public data downloads, and microdata-request forms, are available from the University of Cape Town's DataFirst webpage (65).

### Small-area Demographic and Health Surveillance

Three INDEPTH HDSSs collect longitudinal health and demographic data in three rural surveillance areas. The Agincourt HDSS in the Bushbuckridge district of Mpumalanga province has collected data since 1992 (73); Dikgale HDSS in the Mankweng district of Limpopo, since 1996 (74); and the Africa Centre Demographic Information System (ACDIS) in the Umkhanyakude district in KwaZulu-Natal, since 2000 (75). Agincourt had a population of approximately 90,000 people in 2011 (73); Dikgale, approximately 8,000 in 2008 (74); and ACDIS, approximately 85,000 in 2008 (75).

#### Mortality data collected in and available from HDSSs

Mortality information and a range of other socio-demographic and health information are collected through annual censuses and updates of vital events in each household in the site. Verbal autopsies, a well-established method in the absence of routine death registration, are used for classifying causes of death from population-based inquiries (76–78), and are conducted by specially trained fieldworkers who interview a close relative or caregiver of the deceased. Efforts to refine the approach, have led to international standards for verbal autopsy and strengthening standardised interpretation of verbal autopsy data through the InterVA tool, recently culminating in the launch of the InterVA-4 model (14, 79, 80). At Agincourt and ACDIS, the probable cause of death has been attributed via subsequent physician assessment of the verbal autopsy information (73, 81, 82). However, more recently all three sites have been utilising the automated InterVA tool for probabilistic verbal autopsy interpretation and probable cause attribution (83–85) (e-mail communication from Dr. Chifundo Kanjala and Prof. Marianne Alberts, 4–5 April 2012).

Mortality and population data from ACDIS and Agincourt are available through data products, data downloads, and accompanying supportive documentation at the HDSS’ websites (86, 87). Public access to ACDIS and Agincourt data is available via links to downloadable datasets comprising an approximate 1%- and 10%-sample, respectively, of the full datasets (86, 87). These sample datasets can be used for teaching, familiarising potential users with the structure and availability of data, or developing selected analyses before requesting the full dataset. Unit record data that are not publically available can be requested from senior site staff at ACDIS and Agincourt, accompanied by a motivation, analysis plan, and data user's agreement (86, 87). The INDEPTH Network is committed to the principles and practice of data sharing, and has launched the iSHARE portal aiming to make data from the HDSSs publicly available (13).

### Selected facility-based reporting systems

Apart from South Africa's routine notification system currently reporting incidence and deaths from 33 notifiable medical conditions to local, provincial, and/or national health departments, the country's particularly sizeable burden of disease from cancer, injury, and tuberculosis is reflected in facility-based surveillance systems to help assess the extent and impact of these conditions. Table 3 provides key information about these surveillance systems. Recognising the current international pressure to provide reliable information about maternal and child health to monitor MDGs 4 and 5, and acknowledging the shortcomings in reporting such mortality via civil registration, Table 3 refers additionally to three facility-based structured mortality audits for peri-natal, child and maternal deaths. The value of other national reporting systems, including the South African Birth Defects Surveillance System (SABDSS), South African Dialysis and Transplantation Registry (SADTR), Surveillance of Work-Related and Respiratory Diseases in South Africa (SORDSA), as well as injury-reporting of the Mine Health and Safety Inspectorate, National Transport Information System, and the South African Police Services, are acknowledged but not described here.

**Table 3:** Selected facility-based data sources that may complement vital registration mortality data

Condition- or age-specific data sources		

Programme	Enumeration years	Selected key information about source
Confidential Enquiry into Maternal Deaths (CEMD)	1998–current	Facility-based, structured reporting Compulsory reporting after maternal death has been made a notifiable death Systematic investigation of event, cause and modifiable factors Despite partial coverage, data useful for highlighting main problems and opportunities in addressing maternal mortality Resulted in the publication of two sets of national guidelines*
Peri-natal Problem Identification Programme (PPIP)	2000–current	Facility-based, structured clinical mortality audit of peri-natal deaths Voluntary participation; compulsory in some provinces Data set relates to nearly 3,000,000 births and 108,469 deaths During 2008–09, 275 facilities participated, representing about 963,000 births, i.e. approximately 52% of all facility births for 2008–09 Data not nationally representative, but standardised data collection ensures comparable over time and participating facilities; generate recommendations for better peri-natal care, improved clinical practice; and prioritisation of clinical and public health research.
Child Healthcare Problem Identification Programme (Child PIP)	2005–current	Facility-based, structured clinical mortality audit of paediatric deaths Voluntary participation 2005–2009 data related to 19,295 deaths of 343,408 admissions in 101 hospitals in all nine provinces, representing just under 30% of all hospitals Not nationally representative data, but standardised collection ensures comparable data; Recommendations address key health functions, i.e. policy, management and administration, clinical practice, and education.
National Cancer Registry (NCR)	1986–current	Passive pathology-based surveillance system, with pathology reports confirming a histological cancer diagnosis submitted by selected pathology laboratories Voluntary participation Data obtained from 79 laboratories in 2001 Average of 70,000 cancer cases annually, incl. at least 50,000 new cases
National Injury Mortality Surveillance System (NIMSS)	1999–current	Active collation and centralisation of routinely-kept data of all non-natural deaths entering the forensic medico-legal system at participating mortuaries Voluntary participation Data collection and compilation designed in accordance to particular shortcomings in the national registration system regarding non-natural deaths Systematic information is collected about the incidence and causes of non-natural deaths and demographic characteristics of the deceased 2001–2008: full coverage in a number of large cities
National Tuberculosis Registry (NTR) and Electronic Tuberculosis Register (ETR.Net)	1995–current	Facility-based reporting of case finding, sputum examination, treatment, and outcomes through standardised forms Compulsory reporting – TB is notifiable in terms of the National Health Act Tuberculosis Register (GW 20/11) introduced in 1995 Suspect Register, Laboratory Register, Patient Treatment Card, Clinic/Hospital Card contribute to register Priority reporting by all facilities to DoH within 24 hours for multidrug-resistant (MDR) and extensively drug-resistant (XDR) TB Electronic TB Register (ETR.Net) implemented in 2004/5 – facilitates standardised recording, reporting and evaluation of programmes Information flow: a) Data on case finding, smear conversion and treatment outcomes captured at facility level from patient and facility records; b) collated at sub/district level in electronic TB register; c) data exported into district health information system and transmitted to provincial and national level.

Source: Table created by authors from the following: CEMD: National Committee CEMD, 2008 (91); PPIP: Pattinson (ed), 2011 (92), Bradshaw *et al*., 2008 (93); Child PIP: Stephen *et al*., 2008 (94), Bradshaw *et al*., 2008 (93); NCR: Albrecht, 2006 (95), Mqoqi *et al*., 2003 (96); NIMSS: Matzopoulos, 2002 (97); TB: Dept of Health, 2004 (98), Dept of Health, 2007 (99).

* Note: National Maternity Guidelines for District Hospitals and Clinics, and Essential Steps in the Management of Common Causes of Maternal Deaths in South Africa.

### Secondary data source: ASSA AIDS and Demographic Model

Despite having improved vital registration data and nationally representative HIV prevalence data, these data sources do not provide decision makers with a direct measure of the mortality impact of the country's extensive HIV/AIDS epidemic (56, 100). Mathematical models have hence become necessary, and local actuarial researchers have developed the ASSA AIDS and Demographic Model (101) to estimate such impact. The model has been calibrated to empirical data sources, including vital registration, census, and survey data adjusted for biases (100, 102). As time passed and more relevant empirical data became available, updated revisions of the model were released. A number of mortality and population indicators are available from the models and are widely used as a basis for health policy and planning by both government and the public health research community in South Africa (103–110). While these models are of much practical use, they should be considered ‘interim’ measures until complete vital registration and improved medical certification of causes of death are achieved. Upon online registration, mortality indicators such as _5_ *q*_0_ (under-5 mortality) and _45_ *q*_15_ (adult mortality) are freely available at the website of the Actuarial Society of South Africa (101).

### Adult mortality measures from selected sources

**Fig. 1:**
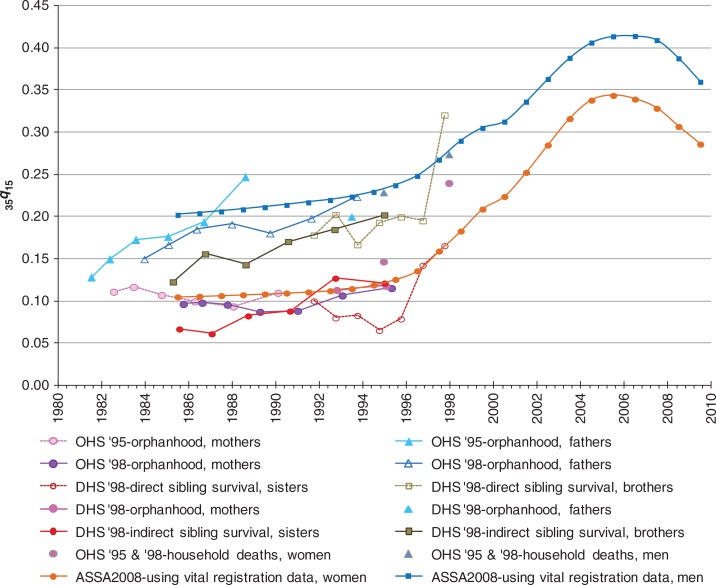
The probability of dying between ages 15 and 50 (_35_*q*_15_) from different data sources.

Source: OHS and DHS estimates extracted from Figure 6.2 in Dorrington RE, Moultrie TA, Timæus IM, 2004 (44). ASSA2008 estimates calculated from ASSA life table values which are based on vital registration data (101).

As this article aims to review mortality data sources and not results from these sources, Fig. 1 is merely provided to indicate the variety of data sources available for estimating adult mortality, specifically the probability of dying between ages 15 and 50 (_35_ *q*_15_). Estimates of _35_ *q*_15_ were derived from using both direct and indirect methods, as indicated in Fig. 1. A fairly consistent trend of increasing mortality for most of the 1990s and early 2000s is produced by the different data sources and methods, with a levelling and decline in mortality in more recent years.

## Discussion

This review demonstrates a rich and varied list of mortality data sources in South Africa. However, it is important to be aware of the strengths and limitations of the different sources and the quality of their data to ensure suitable and strategic utilisation thereof. Different mortality indicators are required for different purposes, and at varied levels of population aggregation. For instance, reliable measures of peri-natal and under-5 mortality at the health-district level are of critical importance in planning and providing for maternal and child health services. Therefore, it is necessary to have robust measures of these indicators at this level, or even at sub-district level. Mortality rates for specific conditions such as tuberculosis, HIV/AIDS, injuries, cardiovascular conditions, neoplasms, and respiratory disease – the high-burden conditions in South Africa – should ideally be measured at magisterial level and at least at health-district level to inform planning for prevention, detection and treatment optimally. Mortality from maternal conditions and other less-prevalent non-communicable diseases, in contrast, can at best be effectively monitored for differences and change at the provincial level, given their relatively infrequent occurrence.

### Under-5 mortality

The under-5 mortality rate (U5MR) is a key indicator of child health and overall development. While its measurement at national level is important for monitoring countries’ progress towards the targets of MDG 4, timely and accurate measurement at sub-national levels are critical for evaluating and prioritising child health care needs and services. Although vital registration is the optimal data source for this, the under-reporting of stillbirths, live births and childhood deaths in South Africa results in under-estimates of child mortality (43, 46, 57). Furthermore, in the context of rapid epidemiological change, the current 2-year reporting delay reduces the utility of the rates. Data from complete birth histories collected in DHSs, are generally a key source for measuring U5MR trends (111, 112), but do not permit estimates lower than provincial/state level. In addition, data quality problems in both the 2001 Census and 2003 DHS have rendered it impossible to derive reliable estimates of under-5 mortality from these sources (113). Census-based summary birth histories may yield estimates at the health-district level, but apart from recall and omission biases, such estimates are limited by their availability only once in 5 or 10 years, and for a reference period of several years preceding the census. Finally, the existing audit programmes for child (Child PIP) and peri-natal (PPIP) events are rich in their content, but biased in that only facility-based events are recorded, and participation in most provinces continues to be voluntarily.

For as long as birth and death registration are incomplete, a strategy is required that would distinguish and integrate useful, quality data from different well-administered sources towards deriving robust data on the levels and determinants of U5MRs at district level. Research in Indonesia, for example, has demonstrated the low-cost, time-efficient potential to adapt the DHS model into a ‘mini-DHS’ to collect data and provide robust under-5 mortality measures at the district level, allowing researchers to point out significant differentials at this level, thus assisting health-district officials to plan for a locally-appropriate response towards achieving national targets for MDG4 (114). Alternatively, South Africa needs to further strengthen the efforts towards complete birth and death registration, a process that has significantly progressed in a short period (57, 115). Particular efforts for children aged 1–4 years are needed (46). The APAI-CRVS (18) initiative and the recommendations of the Health Data Advisory and Co-ordination Committee (109) show great potential for further stimulating vital registration towards completeness.

### Adult mortality levels and causes

Overshadowed by a focus on child health for many decades, it has taken a severe epidemic to modify the neglect of adult mortality in sub-Saharan Africa. This neglect was partly due to the lack of reliable empirical data to measure adult mortality in the region. For most of the past century, South Africa was no exception (41, 116). During the 1980s and 1990s, however, meticulous research efforts started putting together pieces from the disjointed vital registration puzzle (42, 117–119). As alternative data sources became available in tandem with improved civil registration and vital statistics practices, researchers were in a position to triangulate and interrogate different sources and started having a better handle on estimating adult mortality levels (18, 34, 43, 44, 56, 120–124). Differences in adult mortality estimates are shown in these publications, indicating data limitations such as event omission and recall bias in data from censuses and surveys, age misreporting, violation of selected assumptions in indirect methods, and uncertainty about the level of completeness of death registration.

More challenging has been deriving cause-specific mortality estimates. The vital registration system is likely the optimal source to calculate cause-specific estimates from, but a number of problems limit its utilisation, including an incomplete national cause profile due to incomplete death registration, and an urban bias in registration. For reported deaths, limitations of cause data include incomplete medical certification of the cause(s) of death, relatively high proportions of deaths in the ill-defined natural and undetermined unnatural categories, and misclassification of causes of death (104, 110, 117, 119, 125, 126). Cause limitations are exacerbated by the continued practice that traditional headmen, on the basis of relatives’ information about the deaths, are allowed to certify deaths from natural causes. This may affect up to 10% of primarily rural registered deaths (127). While it is a welcome practice in terms of improving completeness of death reporting, it is not ideal for cause-of-death data.

#### Tuberculosis

Alternative sources of causes of adult deaths could be useful for mortality estimates from specific causes. Tuberculosis reporting is compulsory in terms of the National Health Act, and the Register operated by the National Tuberculosis Control Program could play a complementary role, both as a tool to keep track of deaths at the health-district level, and, where data could be matched via linking variables, as a means of assessing agreement of cause attribution between the Tuberculosis Register and vital registration. In addition, this may be useful in assessing the completeness of tuberculosis reporting on the death notification form. The close association between HIV and tuberculosis calls for appropriate strategies to cross-reference data from these two sources for verifying event occurrence and capturing suitable additional data to guide programme improvement to prevent or curb mortality. Similarly, towards optimally informing MDG5, teasing out differing estimates of maternal mortality (109, 128) may gain from linking death records from the Confidential Enquiry into Maternal Deaths and vital registration databases, and triangulation with census data.

#### Cancer

Cancer has a considerable impact on the country's disease burden as the 4th leading category of cause of death in 2000 (104). One in 12 cancer causes, accounting for almost 2,400 cases, were ill-defined and could not be attributed to a site-specific cancer (129), thereby diminishing the utility of the information. It may hence be useful to link records from the Cancer Registry and vital registration databases in a capture–recapture design towards reducing ill-defined cancer diagnoses in the vital registration database. Compared to vital registration data, such registers or audits generally have a considerable advantage in terms of disease control in that they have the potential to measure cause-specific incidence, prevalence, treatment, and case fatality at the health-district or at least provincial level, thus being able to point out differentials at this level, and assisting health-district and provincial officials to identify potential patient load and priorities for locally-appropriate health services.

#### Injuries

For injuries, there is a system problem because the South African death notification form does not include a field for the apparent manner of death (homicide, suicide or accident). All deaths from injuries are certified as ‘unnatural’ deaths, and must undergo a medico-legal investigation at a state mortuary. However, some forensic pathologists consider that, in terms of the Inquest Act, they cannot indicate the circumstance of the death on the death certificate. Thus, the external cause (e.g. burn, firearm discharge, or fall) of many injury deaths is undetermined. While the National Transport Information System records information for selected motor vehicle collision deaths, the Mine Health and Safety Inspectorate records fatal mining injuries, and the South African Police Services violence-related injury, mortality from other external causes is not monitored by any agency (130). The National Injury Mortality Surveillance System (NIMSS) data are filling this gap by providing more comprehensive external cause information which would be valuable in the design and evaluation of injury control programs, but are limited by the lack of full-country coverage and an urban bias (104, 107). For as long as the civil registration data do not include the external cause of injury deaths and NIMSS data do not include all injury deaths, the response to this large cause of premature death and disability can neither be comprehensive nor adequate. It may therefore be worthwhile to adapt the death notification form to include the external cause of injuries, apparent manner of death, scene of injury (e.g. private house, or street/highway), and district of injury (which may differ to the district of death). The value of these data items for injury prevention and safety and peace promotion speak for themselves.

#### HIV/AIDS

Finally, HIV/AIDS was estimated the single largest cause of both death and years of life lost (YLLs) in 2000, respectively, accounting for 30% of total deaths, and 39% of total YLLs (104). Despite its enormous impact on mortality and premature death, HIV is not notifiable in South Africa, and no register or audit are assigned to capture details of suspected or confirmed HIV cases. A number of studies have found HIV/AIDS under-certified in both adult and paediatric deaths (110, 126) (131–134) and although these reports are valuable in alerting data users to problems with the accuracy of cause attribution, they should also be seen as valuable in alerting certifying officers, coders, and researchers to indicator conditions, alternate terminology, and euphemisms that are used to indicate HIV as a possible cause.

A national initiative to improve the quality of medical certification should emphasise the importance of appropriate recording of HIV on death notifications, particularly in the new political climate of acceptance of the role of HIV in causing AIDS (135, 136), towards accurately informing local responses and reliably reporting progress on Target 6A of MDG6 (i.e. have halted by 2015 and begun to reverse the spread of HIV/AIDS). This initiative should be monitored by a medical record review in a representative sample of death notifications to ascertain the veracity of certification and coding practices. Additionally, the HDSSs have built considerable relationships of trust in their communities, and matching HDSS and vital registration records may generate valuable knowledge of the extent of HIV/AIDS misclassification in registered rural deaths. While substantial problems of accuracy have been identified with physician-assigned causes of death in national vital registration data (110, 126), and even in deaths that occurred in tertiary health facilities (39, 132, 133), local HDSS studies using verbal autopsy data have shown successful detection and a substantial presence of HIV-related mortality with closely comparable findings between physician- and InterVA-assessments. (84, 85)

### Challenges and opportunities for mortality measurement

While our review suggests that there are a number of potentially useful data sources on mortality, some of which could be used complementary, it is also clear that data use and analysis based on these collections have been restricted by limitations. At times, mortality data collection has been poor and mortality levels could not be derived due to the extent of missing or illogical data in selected surveys and censuses. For some earlier data sources, the quality of the data was unassessed and the data unused. This may have resulted from a lack of knowledge on how to assess data quality issues, limited capacity to apply selected methods of mortality estimation, prolonged time periods before data become available for public use, financial costs to obtain data, or bureaucratic processes that hinder data access and use. Additionally, sample sizes varied across years for surveys – at times substantially, and not necessarily congruent with national population numbers; the age ranges of respondents for the same data items at times differ across surveys, or across years within a survey; and changing administrative borders and place names have sometimes affected mortality reporting and measurement.

Recognising these challenges presents an important step towards improving mortality measurement, from the planning of enquiry/reporting systems through data collection, processing, and compilation, to depositing data in the public domain for independent evaluation and analysis. Stats SA has greatly improved availability of national mortality data over the past 20 years, has reduced public use waiting time, and has collaborated with strategic partners to improve completeness of vital registration. Mortality measurement will further gain from creating opportunities for wider public knowledge about the importance and public health uses of reliable and valid mortality data; further improvements in completeness of registration and timeliness of data availability; adequate, on-going training of certifiers and coders in cause attribution; and strategic strengthening of analytical capacity at Stats SA and research and academic institutions.

## Conclusion

In a continent often reported as lacking the basic data to infer levels and trends of all-cause and cause-specific mortality, this article has identified a number of data sources in South Africa that, after critical review and adjustment, could yield valuable policy insights into mortality change over the past two decades. Data sources with mortality items are many and varied, offering a promising scenario for improved population health planning from an evidence base informed by multiple sources. However, it is clear that more can and must be gained for mortality measurement by tackling three key issues: data quality, data triangulation, and analytic capacity.

Data quality in surveys and censuses can be improved by demanding nothing less than excellent fieldworker training and excellent quality control measures in the field. For improved quality in vital statistics, further focussed advances in completeness of death registration, and, in particular, a strong, co-ordinated national response towards improved coverage and accuracy of medical certification of causes of death is recommended. The latter is simply critical. Moreover, with studies pointing to problems in physician-certified causes, (137, 138) such causes should not be taken as automatically having content validity, and the possibility of routinely comparing a sample of death certificates with hospital records/doctors’ notes/clinic or day hospital cards, should be pursued.

A focussed agenda is recommended towards data triangulation and contestability via linkage and validation studies that will allow drawing on complementary properties of different sources and, in particular, will assist in completeness estimation and improve our understanding of the accuracy in cause-of-death attribution. Such improved understanding holds clear gains for improved mortality estimates, enhanced resource and service distribution, and, eventually, better meeting the health needs of the population.

However, data quality assessment and triangulation, like other aspects of mortality measurement, require sufficient competent analytic capacity. As analytic capacity has not been expanded upon compared to mortality experiences in the population, nor the increase in national mortality data collection and availability, the expansion and strengthening of analytic capacity is a critical, overarching recommendation.

Achieving these will not be easy, and a co-ordinated research agenda for mortality data collection, evaluation, comparison, analysis, and use, along with an operational agenda for quality assurance and analytical capacity strengthening, are recommended. These should be generated and backed-up by adequate and independent human and fiscal resources. For the future, it will be important to adopt a strategic approach to data collection, streamlined by lessons from past experience, and enhanced by successes and innovative modes of data collection elsewhere.

## Footnotes

1 ‘Population group’ is used as a collective term that may elsewhere be called ‘race’ or ‘ethnic group’. The use of the terms ‘Black African’, ‘Coloured’, ‘Indian/Asian’, and ‘White’ are not intended to denote biological difference, neither to support a racial or ethnic classification system. Under the Population Registration Act of 1950, South Africans were classified into these groups. The classification was associated with disparities in many spheres of life, including health. To acknowledge this impact, and to help track progress in redressing past inequalities based on the classification, mortality and other data are still classified by these terms, although individuals self-classify. For this reason, and because a historical perspective is presented at times, it is necessary to reference these terms in this article.
